# Lower limb maltorsion and acetabular deformity in children and adolescents with X-linked hypophosphatemia

**DOI:** 10.3389/fendo.2024.1422356

**Published:** 2024-09-19

**Authors:** Alexandra Stauffer, Adalbert Raimann, Stefan Penzkofer, Rudolf Ganger, Christof Radler, Gabriel T. Mindler

**Affiliations:** ^1^ Department of Pediatric Orthopedics, Orthopedic Hospital Speising, Vienna, Austria; ^2^ Vienna Bone and Growth Center, European Reference Network Center for Rare Bone Diseases, Vienna, Austria; ^3^ Department of Pediatrics and Adolescent Medicine, Division of Pediatric Pulmonology, Allergology and Endocrinology, Medical University of Vienna, Vienna, Austria; ^4^ MRI Institute Bader, Orthopedic Hospital Speising, Vienna, Austria

**Keywords:** X-linked hypophosphatemia, deformity, maltorsion, magnetic resonance imaging, intoeing

## Abstract

**Background:**

X-linked hypophosphatemia (XLH) is a rare monogenetic skeletal disorder. Lower limb deformities contribute substantially to impaired gait quality and burden of disease in patients with XLH. Standardized data regarding onset and severity of lower limb maltorsion are unavailable. This study aimed to evaluate lower limb maltorsion using rotational magnetic resonance imaging (MRI) and computed tomography (CT).

**Methods:**

Rotational MRI and CT of children and adolescents with verified XLH were evaluated retrospectively. Femoral and tibial torsion, acetabular anteversion, and axial acetabular coverage were measured and compared with published age-adapted radiographic, clinical measurements and MRI data, respectively.

**Results:**

Fifteen patients (mean age, 10.7 years) were included in the study. Decreased femoral torsion was observed in 47% (14/30 femora) and femoral retrotorsion in 17% (5/30 femora). Ten of 13 hips with coxa vara deformity presented with decreased femoral antetorsion. Reduced external tibial torsion manifested in 64% (18/28 tibiae). Abnormal axial femoral head coverage was present in 67% (20/30 hips), whereas 53% (16/30 hips) showed increased acetabular anteversion.

**Conclusion:**

Femoral and tibial torsional pathologies were found in children and adolescents with XLH. The occurrence of severe femoral retrotorsion in a 2-year-old child prior to ambulation raises questions regarding the biomechanical impact of gait on the development of torsional deformities in XLH.

## 1 Introduction

Lower limb deformities, including severe maltorsion, are typical clinical features of children and adults with X-linked hypophosphatemia (XLH, OMIM 307800). Musculoskeletal pain, gait deviation, deformities of the lower limbs, and short stature result from chronic hypophosphatemia caused by a loss of function of a phosphate-regulating gene with homology to endopeptidases on the X chromosome (1).

Musculoskeletal symptoms heavily impact quality of life, thus contributing to the burden of disease, and have been reported by a majority of patients with XLH. Pain, mainly joint or bone pain reported by 80% of children and muscle pain reported by 60%, was among the predominant symptoms. In addition to joint stiffness, impaired gait consisting of delayed walking, unusual gait or way of walking and/or running, and the use of walking devices have been reported (2). These findings were further analyzed by our study, which confirmed gait abnormalities and lower limb deformities in patients with XLH, with significantly increased lateral trunk movement and the combination of complex frontal, sagittal plane, and torsional deformities (3–5).

The correlation between gait analysis and torsional computed tomography (CT) was evaluated in previous studies reporting a strong correlation between the anatomic tibial torsion angle measured by CT and gait data (knee rotation). Regarding the femur, however, low correlation with gait was observed (6, 7), thus necessitating torsional magnetic resonance imaging (MRI) for sufficient torsional deformity analysis; gait analysis alone poorly represents bony features of the femur.

Details regarding onset and severity of tibial and/or femoral maltorsion have not been reported thus far. This study aimed to quantify lower limb maltorsion and acetabular deformities in children and adolescents with XLH using torsional MRI.

## 2 Materials and methods

A single center retrospective analysis of torsional MRI in a pediatric and adolescent cohort with verified XLH was conducted. The study was approved by a local Ethics Committee. Because of the retrospective study design, no written informed consent was obtained. The imaging database was reviewed for torsional CT and MRI of pediatric patients with XLH. Between 2010 and 2021, data regarding 16 children and adolescents were available. Incomplete scans and segments that had undergone previous osteotomies were excluded from further evaluation. Thus, data of 15 children was used for further evaluation, as one child had undergone surgery on both femora and tibiae.

### 2.1 MRI and CT analysis

Torsional MRI of children and adolescents was performed at our institution using a 1.5 Tesla MRI with the following protocol: transverse imaging, supine placement of patient with legs extended and parallel to the x-axis of the scanner, T2-weighted turbo-spin-echo, section thickness of 4 to 5 mm, head matrix coil, 25 sections for each region, full body landmark localizer for the femoral neck, including entire femoral head and lesser trochanter, the knee joint, and talocrural joint imaging. To minimize movement artifacts, lower limb constraints, such as weighted blankets, were used if necessary. For the youngest child (2 years old), torsional MRI to assess delayed onset of walking was performed with the patient sedated as part of scheduled cerebral MRI. All other imaging procedures were completed without the need of sedation or anesthesia.

Non-contrast-enhanced torsional CT of the hips, knee, and ankle was performed between 2010 and 2014 in only 3 cases. Torsional CT is not commonly performed at our clinic because of concerns regarding radiation exposure in a younger patient cohort.

**Figure 1:**
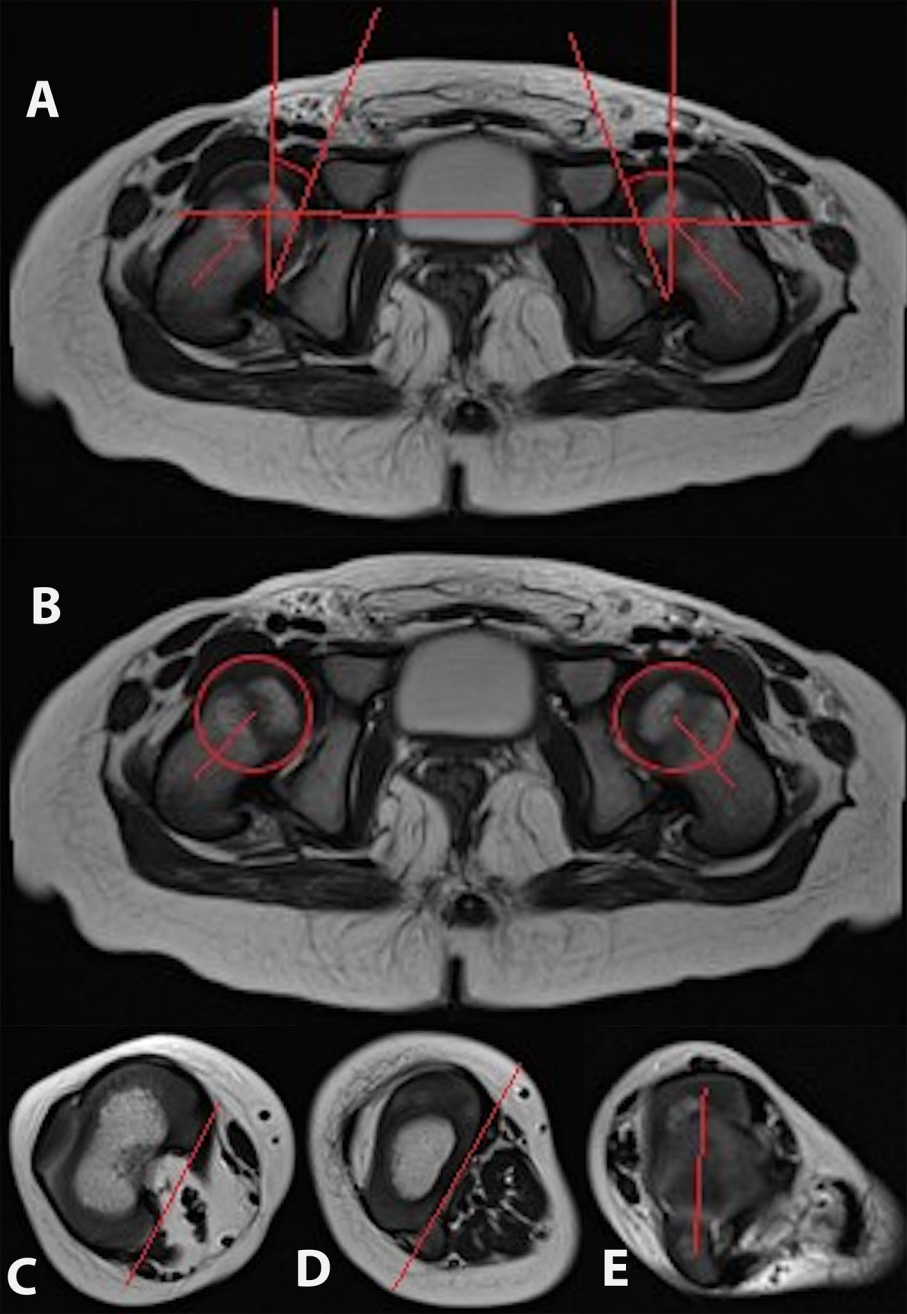
Axial/frontal MRI sections with measurement and reference lines of acetabular anteversion **(A)**, acetabular coverage **(A)**, proximal femur **(B)**, distal femur **(C)**, proximal tibia **(D)**, and bimalleolar axis **(E)**.

Imaging analysis was performed by a senior radiologist specializing in lower limb imaging at our institution. Femoral and tibial torsion was measured according to a modified Hernandez method (8). Analysis of femoral torsion is performed by measuring the angle between a line connecting the femoral head center with the midpoint of the base of the femoral neck superior to the lesser trochanter with a line parallel to the posterior surface of the femoral condyles (9). Tibial torsion is measured by determining the angle between a reference line drawn tangent to the posterior aspect of the tibial plateau in the first image section just below the articulating surface of the knee where the tibial plateau is fully visible and another line drawn bisecting the medial and lateral malleoli, just above the articulating surface of the ankle where both malleoli are most prominent (10). Acetabular anteversion angle (AAA) was defined as the angle between the bony posterior and anterior margins of the acetabulum and a reference line perpendicular to a line connecting the triradiate cartilage or center of the acetabulum of both sides. The axial total coverage angle was defined as an angle between the bony anterior and posterior margin of the acetabulum and the center of the femoral head (**Figure 1**). Measurements were made on axial view images at the level of highest femoral head circumference and were compared with published MRI data regarding healthy children (11). Femoral torsion was compared with age-adapted historical radiographic measurements (12),whereas the comparison of tibial torsion was with age-adapted historical clinical values (13) because of the lack of MRI normal values in the literature. Previously published MRI data regarding femoral and tibial torsion in 7 children (5) were included in this study.

### 2.2 X-ray analysis

The neck-shaft angle (NSA), defined as the angle between the femoral neck axis and the femoral shaft axis, was measured from routinely obtained long leg standing radiographs within a month of the torsional MRI (with the exception of our youngest patient) by determining the angle between a line from the middle of the femoral head intersecting the medial and lateral border of the femoral neck and a line midway between the lateral and medial borders of the femoral shaft (14).

## 3 Results

Fifteen children and adolescents with an average age of 10.7 years (age range, 2 to 19 years; female:male ratio = 11:4) were included in the study. Eight participants received conventional therapy consisting of orally administered phosphorus and vitamin D, whereas 7 underwent burosumab therapy (**Table 1**). The mean time of burosumab administration was 13 months (between 3 and 22 months). MRI data regarding 12 children and adolescents, and CT data regarding 3 were analyzed for transverse deformities of the lower extremities. One patient was excluded because of previous osteotomies of both femora and tibiae. Another had undergone distal osteotomy of the tibiae to correct increased internal torsion and varus deformity; therefore, only femoral data were included for evaluation for this patient. No participant had undergone previous surgical treatment of the hip or spine. Seven (47%) of the 15 patients had undergone guided growth procedures using tension plates and screws before torsional imaging was obtained. All femoral, tibial, and acetabular values obtained using torsional MRI and CT in this cohort are listed in **Table 2**.

**Table 1:** Average dosage for 8 children and adolescents receiving conventional therapy.

Oral medication	No. of patients	mg/kgBW	Standard deviation
Calcitriol*	8	0.026	0.014
Phosphorus	8	57.8	51.1
Burosumab†	6	1.25	0.45

*1 µg of alfacalcidol was calculated as 2 µg of calcitriol. †Seven children underwent burosumab therapy at the time of magnetic resonance imaging. Data regarding 1 participant were not available from the rural outpatient center at which the patient was treated.

**Table 2:** Femoral and tibial torsion values obtained by torsional magnetic resonance imaging or computed tomography compared with age-matched normal values*.

Patient	Sex	Previous surgery	Age (yr)	Femoral torsion		Tibial torsion		Acetabular anteversion		Acetabular coverage		Neck-shaft angle	
(degrees)													
				R	L	R	L	R	L	R	L	R	L
XLH 1†	M	None	5.4	+27	+1	−3	−1	22	21	144	150	136	128
XLH 2†	F	None	10.8	+2	+1	−3	−1	18	14	142	146	115	119
XLH 3†	F	GG	8.5	−15	+2	−9	−4	14	17	155	149	120	129
XLH 4†	F	GG	8.7	+14	+11	+3	+6	17	16	158	153	133	134
XLH 5†	M	GG	16.1	+8	+1	−4	−11	10	6	150	139	123	124
XLH 6†	F	GG	11.6	+17	+14	−1	+5	16	16	146	151	131	129
XLH 7†	F	None	8.9	+19	+21	−11	−5	12	13	158	132	136	140
XLH 8	F	GG,osteotomy	10.2	+27	+16	−	−	16	13	142	133	141	137
XLH 9	F	None	11.4	+20	+29	+1	+6	17	15	146	136	143	147
XLH 10	F	None	14.8	+14	+14	+2	+4	19	19	138	140	130	136
XLH 11	F	None	18.2	+24	+8	+1	+10	19	20	155	166	145	134
XLH 12	F	None	19.8	+11	+15	+14	+16	18	23	152	150	129	129
XLH 13	F	None	2.0	−11	−5	−28	−22	17	22	122	130	127	130
XLH 14	M	GG	7.6	−1	+11	−7	+4	18	18	142	134	119	124
XLH 15	M	GG	7.1	+11	−13	+12	+8	7	10	130	125	110	116

*Historic normal graphs were used for age-matched comparison. Therefore, femoral positive values represent internal torsion and negative values represent external torsion. Tibial values are vice versa, with negative values representing internal torsion and positive values representing external torsion. †Patient received burosumab therapy at the time of rotational MRI with a mean treatment time of 13 months. Femoral and tibial values are part of previously published data (5).5 GG, guided growth; R, right; L, left; M, male; F, female; XLH, X-linked hypophosphatemia; red values, reduced femoral or tibial torsion or acetabular anteversion or coverage; blue values, increased findings compared with age-matched normal values; –, data regarding 2 tibiae of 1 patient were excluded because of previous derotational surgery. XLH 4, 6, 9 and 12 are relatives from one family, respectively, XLH 13 and 14 from another.

### 3.1 Acetabulum

**Figure 2:**
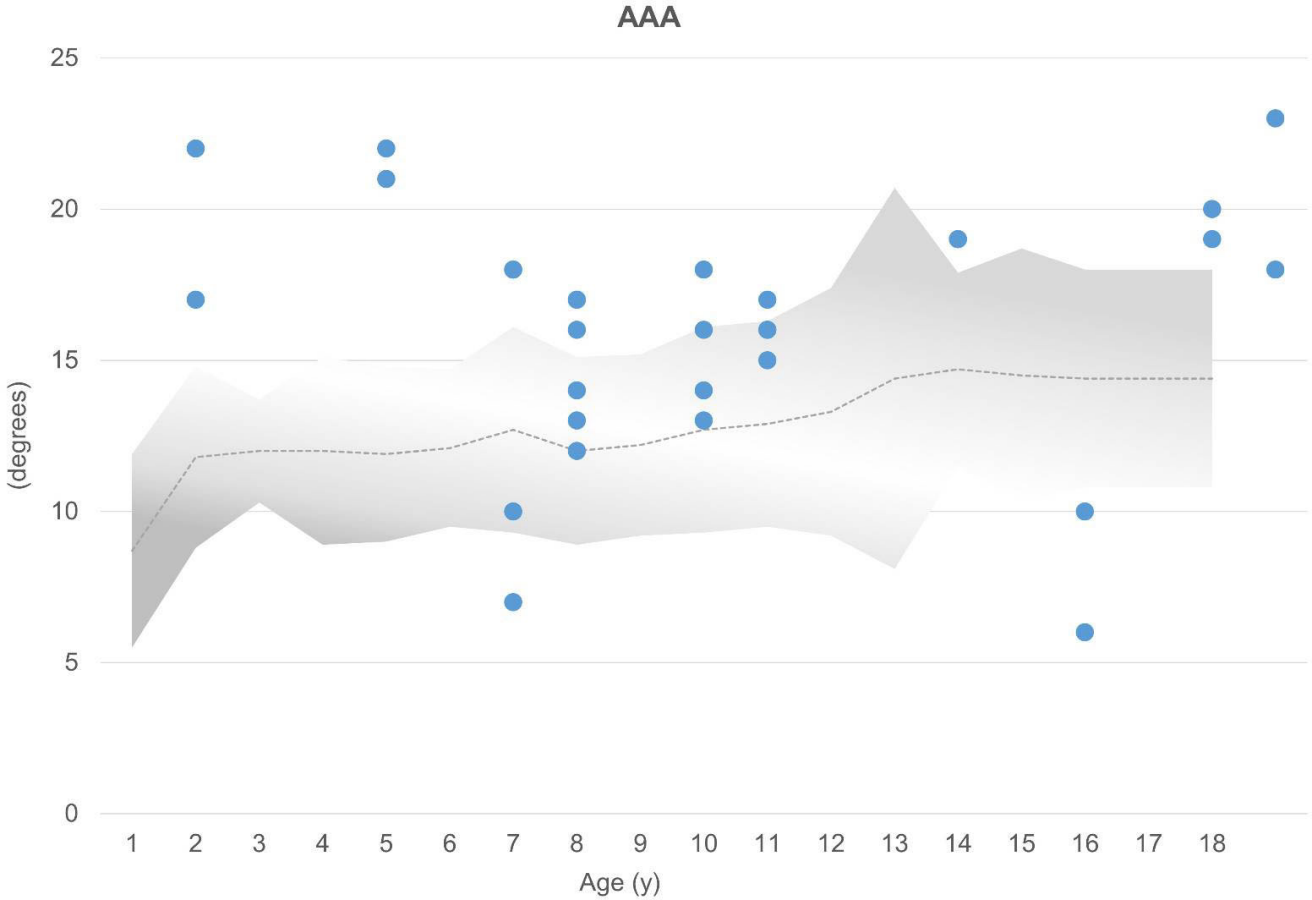
AAA (above) are compared with age-matched normal values (gray area). Normal mean values are represented by a dotted line (11). Each acetabulum is represented by a dot (n = 30). Above gray area, increased AAA; below gray area, decreased AAA.

**Figure 3:**
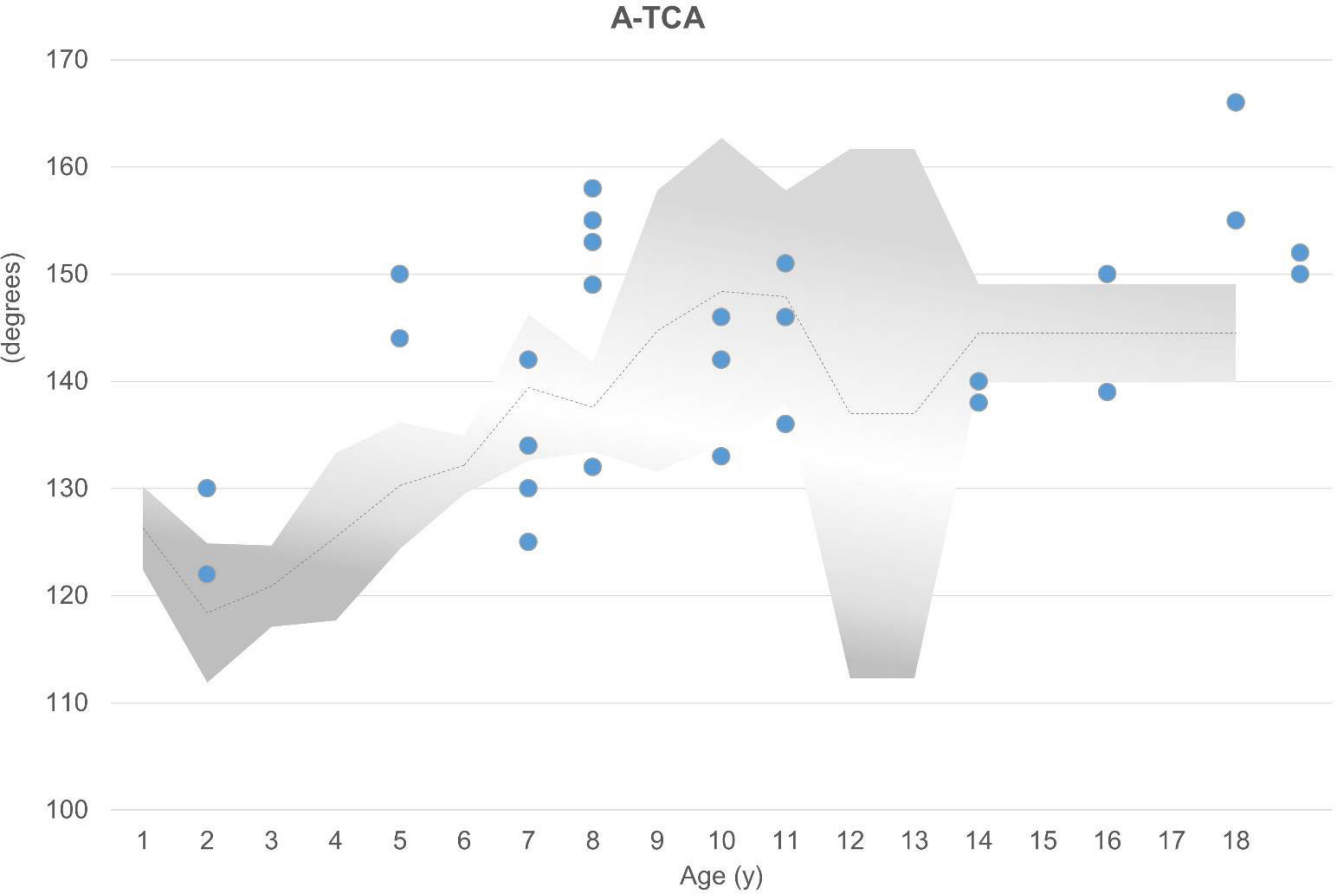
Slightly increased a-TCA compared with age-matched normal values (gray area; mean shown by dotted line) was observed in 13 of 30 hips, represented by the dots above the gray area (11). Seven hips had decreased TCA.

The mean AAA was 16 degrees (n = 30; range, 7 to 23 degrees). Increased AAA was observed in 16 (53%) of 30 hips, whereas 3 acetabula showed decreased anteversion (**Figure 2**). The majority (67%, 20 of 30 hips) showed abnormal values regarding the axial section total femoral head coverage angle (a-TCA), with 13 of 30 hips presenting with increased a-TCA ranging from 122 to 166 degrees (**Figure 3**). Seven hips had reduced coverage. All values were compared with healthy age-matched controls in the literature (11).

### 3.2 Femoral torsion

**Figure 4:**
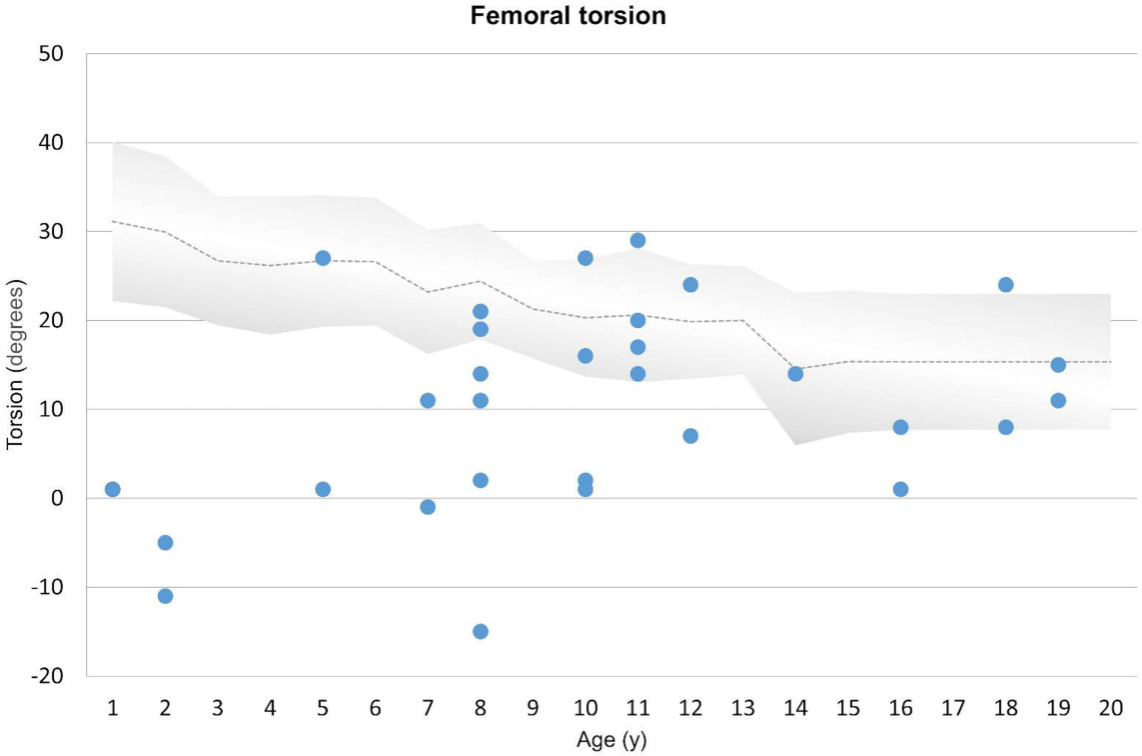
Representation of femoral torsion compared with age-matched historical radiographic normal values (gray area) (12). Course of torsional changes, ranging from internal (positive values) to external (negative values) in relation to age is shown above. Each femur is represented by a dot. Decreased anteversion and severe pathological changes (retroversion) are marked below the gray area.

Overall, femoral internal torsion was reduced by a mean of 10 degrees, ranging from internal torsion (antetorsion) of 29 degrees to external torsion (retrotorsion) of 15 degrees. Furthermore, 14 (47%) of 30 femora showed reduced internal torsion compared with age-matched control groups in the literature (12). Severe pathological femoral torsion (femoral retrotorsion) was observed in 5 (17%) of 30 femora ranging from 1 to 15 degrees. A 2-year-old girl with delayed onset of walking presented with retrotorsion of both femora with values of 5 degrees external torsion in the left femur and 11 degrees in the right. One femur of an 8-year-old girl showed external torsion of 15 degrees external torsion and a reduced antetorsion of 2 degrees internal torsion (**Figure 4**).

### 3.3 Coxa vara

The mean NSA was 130 degrees, ranging from 110 to 147 degrees (15). The presence of coxa vara was observed in 13 (43%) of 30 hips, and coxa valga was observed in 3 hips (10%). Coxa vara was associated with reduced torsion of the femur in all except 3 hips. However, torsional values were in the lower normal range in those cases. In all cases of increased femoral torsion, coxa valga was present (**Figure 1**).

### 3.4 Tibial torsion

**Figure 5:**
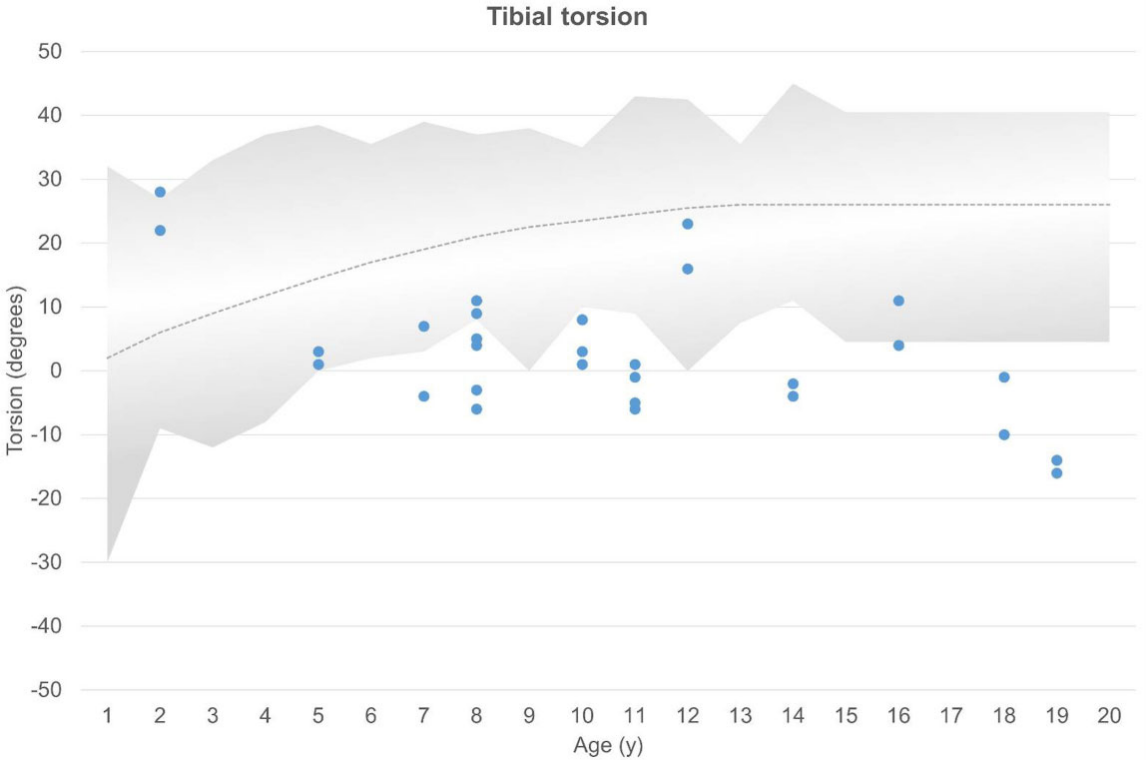
Tibial torsion compared with clinical age-matched normal values (13). External and internal torsion and age are shown on the axes. The gray area shows normal values, with each dot representing 1 tibia. Decreased tibial torsion is seen below the gray area.

Tibial torsion ranged from 16 degrees internal to 28 degrees external, with an overall mean external torsion of 1 degree. Decreased tibial torsion was observed in 18 (64%) of 28 tibiae, and lower normal range was observed in 4 of 28 tibiae according to age-matched clinical normal values (13) (**Figure 5**).

The combination of pathological tibial and femoral torsion was examined to determine the presence of possible compensatory mechanisms. However, no significantly increased tibial torsion in combination with decreased femoral torsion, and vice versa, was observed.

## 4 Discussion

XLH-associated deformities quantified by gait analysis and frontal plane radiological deformity analysis were previously reported in the literature (3–5, 16). Maltorsion of the tibia associated with XLH was suspected or measured in recent studies (3–5, 17, 18). However, data regarding femoral and acetabular changes are limited (17, 18) and data regarding onset of these deformities are lacking.

In this study, torsional data regarding 15 pediatric patients with XLH obtained by MRI or CT showed maltorsion of the tibia with pathological values compared with age-matched controls in 68% and pathological femoral torsion in 57%. In line with recent literature (17, 18), acetabular anteversion and coverage changes were observed using MRI and CT.

### 4.1 Acetabulum

Bonnet-Lebrun et al. (18) found normal acetabular anteversion in 41% of cases and normal acetabular coverage in 72% using EOS imaging (EOS Imaging, Paris, France) in children and adolescents with XLH. However, our study showed normal acetabular anteversion in 37% and normal acetabular coverage in 33% as compared with age-matched normal values (11).

Based on CT findings, Scorcelletti et al. (17) reported significantly (P < 0.05) increased acetabular coverage and increased acetabular version in adults with XLH. However, for the interpretation of acetabular coverage angles in adults with XLH, the early onset of osteoarthritis as a common and typical problem in adults with XLH has to be considered. Osteophytes at the acetabular can significantly increase adult values in XLH cohorts.

### 4.2 Femoral torsion

Femoral torsion has been reported to increase intrauterine (19). Torsional abnormalities can be related to intrauterine position of the legs, considering that an abnormal degree of internal rotation of the leg leads to increased femoral torsion and that an increase in external rotation results in retroversion (19). Developmental changes of normal anteversion of the femoral neck are well documented in the literature, derived from historical clinical and/or radiographic studies (12, 19–21). These results are widely accepted and used as reference values to determine pathology. Femoral anteversion steadily decreases during childhood until completion of growth (12, 20, 21), whereas external tibial torsion increases until a mean of 25 degrees (13). Therefore, it is necessary to compare pediatric torsional data with an age-matched control group to identify true deformity and to distinguish between normal development and disease-related deformity.

Bonnet-Lebrun et al. (18) examined 35 children with XLH between the ages of 5 and 14.5 years with EOS imaging, which suggested a wide range of torsional and planar deformities compared with the control group. Subgroup analysis of frontal malalignment, including genu varus and genu valgus, was performed. Femoral torsion and tibial torsion were divided among the subgroups. Although comparison of the data with normal group values was performed, the impact of the natural course of femoral and tibial torsion might have been underestimated.

The youngest child included in this study showed retroversion of - 11 degrees in the right hip and - 5 degrees in the left at age 2 years before the onset of walking, thus challenging the theory of body weight as a biomechanic impact on the development of lower limb deformities and implicating a dysplastic component of XLH. Our patient underwent torsional MRI as part of routinely scheduled cerebral MRI to exclude the presence of cranial synostosis and Chiari I malformation occurring with a high incidence in patients with XLH (22). To our knowledge, femoral maltorsion (decreased femoral internal torsion) before the onset of ambulation in infants has not yet been reported in cases of XLH. We recently formulated our theory of the dysplastic characteristic of XLH being caused by early onset of torsional deformities, which can occur independently of radiographic signs of rickets (5). The case of deformity onset before gait again challenges the theories of rachitic (hypophosphatemic) changes and pure biomechanical loading as the causes for the development of lower limb deformities in children with XLH.

Femoral maltorsion has been recently suspected (3, 4) and identified (5, 17, 18) in children and adults with XLH. However, this is the first study to describe severe femoral maltorsion in comparison with a normal age-matched group. As noted above, femoral torsion decreases during growth as a natural course, which is an overall development that was not observed in this XLH cohort.

### 4.3 Coxa vara

Radiographic normal values using the Dunlap method for determination of femoral torsion are widely available. Among the earliest, Shands and Steele (20) conducted a 4.5-year follow-up and reported graphs for the average normal femoral torsion and the average normal femoral neck angle during growth in 238 healthy children between 3 months and 16 years of age. Patients with coxa vara presented with decreased femoral torsion, and even cases of retroversion were observed.

Comparing the findings of Shands and Steel (20) with our study cohort, we observed coxa vara in children with reduced femoral antetorsion or lower normal values. All cases with retroversion presented with coxa vara, whereas all hips with increased femoral antetorsion were associated with coxa valga.

### 4.4 Tibial torsion

Intoeing gait is one of the most common problems seen by pediatric orthopedists and can be caused by increased internal tibial torsion. Internal rotation of the great toe occurs from an initial preaxial position into the final medial position *in utero*. Staheli (13) clinically examined 1000 legs of children and adults to establish normal values for tibial torsion during growth. The authors observed a steady external rotation of the tibia during growth until the normal range (0 to 45 degrees; mean, 25 degrees) was achieved during middle childhood. Most tibial torsional problems in normal developing children resolve with age. However, with persistent increased internal torsion of more than 15 degrees associated with substantial functional impairment, derotational tibial osteotomies at a distal level might be indicated (23, 24).

Similar to other reports (5, 17, 18) and in accordance with previous gait analysis studies (3–5), we observed reduced tibial torsion in cases of severe internal maltorsion. Furthermore, the typical postnatal change of tibial torsion during growth (internal to external) was not observed in our XLH cohort.

Lower limb maltorsion has a strong impact on the quality of radiographic disease assessment in children with XLH, which should be considered when planning deformity correction surgeries. First, difficulties in obtaining adequate full-length standing radiographs in the anteroposterior (AP) projection can occur because of severe maltorsion. Directing the patellae forward while keeping both knee joints in a neutral position allows for parallel orientation of the femoral condyles to the x-ray film, thus enabling correct evaluation of the lower limb alignment (25). Second, Thacher scoring of rickets depends partly on radiographic technique. Thacher et al. (26) noted that because of rotation of the femur or tibia, the physis might appear to be partially lucent and to have a smooth visible metaphyseal margin, thus mimicking the mildest grade of rickets. Assessment of femoral or tibial torsion should be ascertained sufficiently to prevent misjudgment of the severity of rickets.

The widely accepted method for torsional deformity analysis of the lower limbs without radiation is MRI. Recent studies evaluated lower limb torsion using the EOS imaging system, which acquires low-dose standing radiographs in both frontal and sagittal planes simultaneously and allows for 3D reconstruction of the images. It is comparable to rotational CT of children and adolescents with the benefits of considerably reduced radiation (27), and additional information is available because of the weight-bearing character of the images. The efficacy of the EOS imaging system compared with 3D CT and MRI axial imaging was analyzed by Brooks et al. (28) The authors found comparable results with tibial torsion. However, low-dose biplanar femoral values were significantly (P < 0.001) lower than those of CT and MRI.

Torsional abnormalities of the femur and the tibia are associated with in- or out-toeing in children with a higher risk of stumbling, impaired gait (5), and pain primarily in the hip and knee (29). In adults, these abnormalities present as hip pain caused by femoroacetabular impingement, anterior knee pain, and patellar instability and are associated with a higher risk for developing osteoarthritis in the affected joint (30, 31). Considering that XLH is known for early onset of osteoarthritis, further studies are needed to analyze the impact of maltorsion on the development of osteoarthritis of the hip, knee, and ankle.

We frequently perform surgical derotation of the distal tibia, both acute and gradual, to correct internal foot progression in children and adolescents with XLH, severe maltorsion, and a high burden of disease because of stumbling. However, we have rarely addressed femoral maltorsion. Furthermore, femoral maltorsion and acetabular deviations can contribute to femoroacetabular impingement, which has not been described to have been addressed in cases of XLH. We warn of surgical correction of all aspects of lower limb deformities without adequate previous deformity assessment of the whole lower limb (acetabulum to ankle) in patients with XLH. It is yet to be determined to what extent the recently described deformities require surgical intervention in children with XLH.

In our study, no patient had combined femoral, tibial, and acetabular values within the normal range. Compared with a high rate of 92% of children presenting with frontal plane deformities (3), this indicates torsional abnormalities to be a predominant occurrence in children and adults with XLH.

### 4.5 Endocrinological considerations

From an endocrine perspective, lower limb maltorsion is a crucial factor for functional outcomes and quality of life, as evidenced by the strong association between gait quality and physical QoL (1–4, 16). Despite the widely recognized importance of lower limb deformities in the disease burden of XLH patients, the evaluation of these deformities has been underrepresented in academic research until recently. Current understanding remains limited regarding which specific disease- or treatment-related factors most significantly influence the progression of these complex deformities. Variations in phosphate metabolism and hormonal imbalances characteristic of XLH may play pivotal roles, yet their precise impact on maltorsion progression is not well-defined. Additionally, the interplay between genetic predispositions and biomechanical forces during growth and development requires further exploration.

### 4.6 Limitations

This study has various limitations. Our cohort is heterogenous; we decided to include all children and adolescents who received torsional imaging with or without previous guided growth procedures (only bony osteotomies were excluded). However, because of the rarity of this disease, only limited numbers in each age group were available for evaluation. The fact that nearly half our patients underwent guided growth procedures before torsional MRI or CT might impact the accuracy of this study, considering that temporary hemiepiphysiodesis might not affect only frontal plane correction. Furthermore, this study did not include gait analysis data. Therefore, analysis of a direct relationship between gait deviations and torsional deformities in this cohort with XLH was not possible. All measurements were performed by 1 senior radiologist to minimize interobserver bias. However, this leaves intraobserver reliability as a limitation of the study. Determination of femoral torsion angles can be obtained by various methods. However, no generally accepted definition exists. Measuring methods differ regarding the anatomic landmark selection for the reference line determining femoral neck axis and can be distinguished into 2 groups: transverse section and oblique section methods (9, 17). Most CT and MRI measuring methods compare their findings with each other, with only a few earlier ones referencing historical normal values of torsion obtained either clinically or radiographically (8, 32–34). For analysis, we used a modified Hernandez method (8) measuring the femoral axis parallel and in the middle of the neck and the distal line at the posterior medial and lateral femoral condyles. However, from a biomechanical view, the most relevant axis is yet to be determined (17). Our measurements were focused on total femoral and tibial torsion. Differentiation between intertrochanteric, femoral shaft, and condylar maltorsion, as previously described (17), was not performed. The NSA was measured from long leg standing radiographs; no additional pelvic (AP view) radiographs were obtained, potentially rendering less accurate measurements of NSA. Furthermore, compared to long leg standing x-rays, hand x-rays are not routinely obtained at our clinic during follow-up and were not available for evaluation at the time of the MRI/CT. Therefore, Thacher score for rickets evaluation was not performed due to incomplete imaging (26). A correlation between the pharmaceutical treatment options and development of complex deformities could not be established with this small cohort due to several limitations. Further studies are needed to adequately evaluate the impact of therapeutic agents on deformity progression and development.

## 5 Conclusions

This study revealed femoral torsional pathologies with early onset of XLH. We observed femoral retrotorsion in a child as young as 2 years before verticalization and weight-bearing. All cases with retroversion presented with coxa vara. Further torsional studies in young children with XLH are needed for a sufficient evaluation of onset and development of lower limb deformity (acetabulum to ankle) and the impact of new medical treatments on this deformity development should be analyzed.

## Data Availability

The raw data supporting the conclusions of this article will be made available by the authors, without undue reservation.
